# Rectus sheath patch. A novel surgical technique in the repair of isolated renal pelvis necrosis in a transplanted kidney. A case report

**DOI:** 10.1016/j.ijscr.2024.110026

**Published:** 2024-07-10

**Authors:** Sebastian Primrose, Ai Lin Tan, Malcolm Lawson, Handoo Rhee, Anthony Griffin

**Affiliations:** Queensland Kidney Transplant Service, Princess Alexandra Hospital, Brisbane, Queensland, Australia

**Keywords:** Urine leak, Renal transplant, Rectus sheath patch, Necrotic renal pelvis

## Abstract

**Introduction:**

Focal necrosis of the renal pelvis in a transplanted kidney is a rare but often morbid complication that may lead to graft loss. Given the scarcity of donor organs, all attempts are made to preserve the graft. Currently there is no standard surgical technique for reconstruction or repair of isolated renal pelvic necrosis.

**Presentation of case:**

A 70-year-old male with end stage kidney disease underwent renal transplantation. The patient developed a day-three post-operative urine leak. During surgical exploration, a focal area of pelvic necrosis was observed without evidence of proximal or distal ureteric involvement. Given the excellent function of the renal allograft, a novel surgical technique was successfully used to repair the necrotic defect. Reconstruction of the renal pelvis was performed using an avascular rectus sheath patch. The patch was secured over the open pelvis following necrotic tissue debridement. The patient made a successful recovery with complete resolution of urine leak. A 6-week post-operative retrograde pyelogram confirmed no ongoing urine leak.

**Discussion:**

To restore anatomy, the pelvic defect was patched with avascular rectus sheath fascia. Advantages of this reconstructive method were technique simplicity and low donor site morbidity. Potential complications included patch failure with ongoing urine leak, ventral wall hernia through the fascial donor site and stenosis of the ureteropelvic junction.

**Conclusion:**

This case highlights the successful surgical management of a renal pelvis urine leak patched with rectus sheath fascia. This technique could be considered as a graft saving procedure in similar cases where the alternative is transplant nephrectomy.

## Highlights

• Successful repair of an isolated urine leak using a rectus sheath patch
• Urine leak following renal transplantation
• A novel surgical technique in the repair of an isolated renal pelvis urine leak

## 1 Introduction

Kidney transplantation remains the treatment of choice for most patients with end stage renal disease. It offers significant survival benefit and improved quality of life compared to maintenance dialysis [1]. Surgical complications in the immediate post-transplant period are a relevant problem and can be broadly divided into vascular, urological and others such as wound healing issues. The incidence of urological specific complications following transplantation range between 3.7 % to 15 % [2,3]. A major cause of such is devascularisation and subsequent tissue necrosis typically manifesting as urine leakage. Urine leaks may present in the immediate or early post-transplant period (up to 3 months) and are associated with significant patient morbidity, including potential graft loss and even mortality [4]. It is usually the result of ischemic necrosis of the distal ureteral segment of the ureterovesical anastomosis [5]. Typically, urine leaks present with progressive abdominal pain, increasing serum creatinine and high surgical drain output. Diagnosis is confirmed with a high fluid/serum creatinine ratio with cross-sectional imaging often demonstrating a fluid collection [5,6]. Although endourological interventions including bladder catheterisation, percutaneous nephrostomy and ureteral stent placement may be sufficient in cases of minor leakage, surgical reintervention is often required. Open surgical approaches such as ureteral reimplantation are standard practice [6]. If necrosis extends to the proximal ureter or distal renal pelvis, reconstructive pyeloureterostomy with native ureter may be considered [7].

Necrosis isolated to the renal pelvis without distal or proximal ureter involvement is rare and challenging to treat. Occlusion or inadvertent ligation of an aberrant polar or segmental artery during organ recovery, bench dissection or implantation may be the cause.

For such an uncommon condition, there are no standardized techniques for renal pelvis reconstruction or repair in the case of isolated necrosis in transplanted kidneys. In this report we describe a 70-year-old patient who developed a urine leak secondary to renal pelvis necrosis following kidney transplantation. A novel surgical technique using an avascular rectus sheath patch was attempted with a successful outcome for the patient. This case has been reported in line with the SCARE criteria [8].

## 2 Case presentation

A 70-year-old male patient with end stage renal failure due to polycystic kidney disease received a kidney allograft at the Princess Alexandra Hospital. The patient had been on home haemodialysis for 3 years via a right forearm radio-cephalic fistula, had a residual urine output of 500 mL/day with a dry weight of 97 kg. His medical history was significant for well controlled hypertension, class II obesity and non-melanotic skin cancer. A pre-operative echocardiogram showed an ejection fraction of 52 % with normal biventricular function.

The allograft was recovered from a 45-year-old decreased donor with a baseline creatinine of 87 μmol/L (umol/L)**.** The Kidney Donor Profile Index (KDPI) was 83 with an Estimated Post-Transplant Survival Score (EPTS) of 93 %. The graft had standard vascular and ureteral anatomy. The cold ischaemic time was 8 h ours with a warm ischaemic time of 28 min. End-to-side vascular anastomoses were performed onto the external iliac vein and artery followed by a dome ureteroneocystostomy over a 7 French (fr) double pigtail stent. Initial perfusion was excellent with urine production on table. A 15fr Blake's drain was left in-situ. The patient was commenced on a standard immunosuppressive protocol consisting of mycophenolate, tacrolimus and dexamethasone.

A day-one post-operative ultrasound confirmed a well perfused kidney with appropriate vascular resistive indices. A serum creatinine drop of 119 μmol/L was noted with a consistent urine output of 100–150 mL/h. On the third post-operative day, urine output had significantly decreased to 10 mL/h before the patient became anuric with an associated 100 μmol/L rise in serum creatinine over 24 h. Drain output was measured at 100 mL/h of straw-coloured fluid. The patient described severe constant abdominal pain overlying the graft. Focal right lower quadrant tenderness with associated guarding was noted during physical examination. A urine leak was suspected, and a drain fluid creatinine of 3000 μmol/L was confirmed. An urgent ultrasound demonstrated an anechoic, non-septate fluid collection surrounding the renal graft with maintained perfusion and appropriate vascular indices. The combination of new onset abdominal pain, significantly elevated drain fluid creatinine and an ultrasound demonstrating a fluid collection was highly suspicious for a urine leak. The patient was urgently taken to the operating theatre for surgical exploration.

**Fig. 1:**
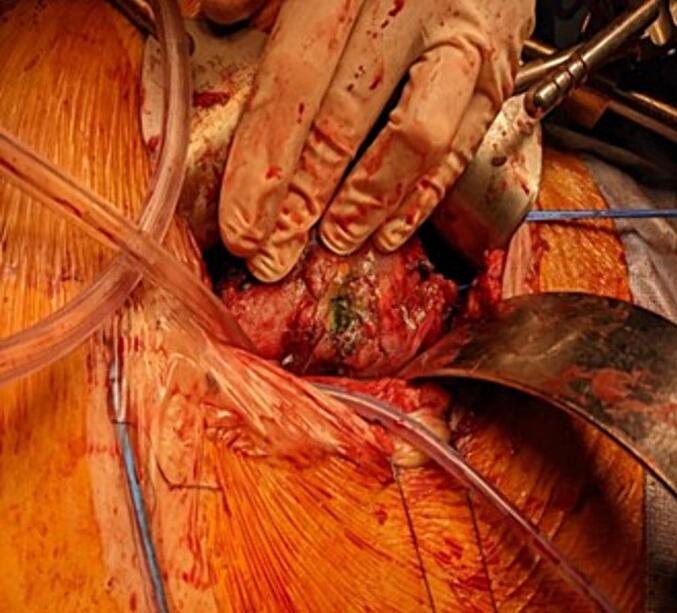
Intraoperative photo demonstrating focal necrosis localised to the renal pelvis.

**Fig. 2:**
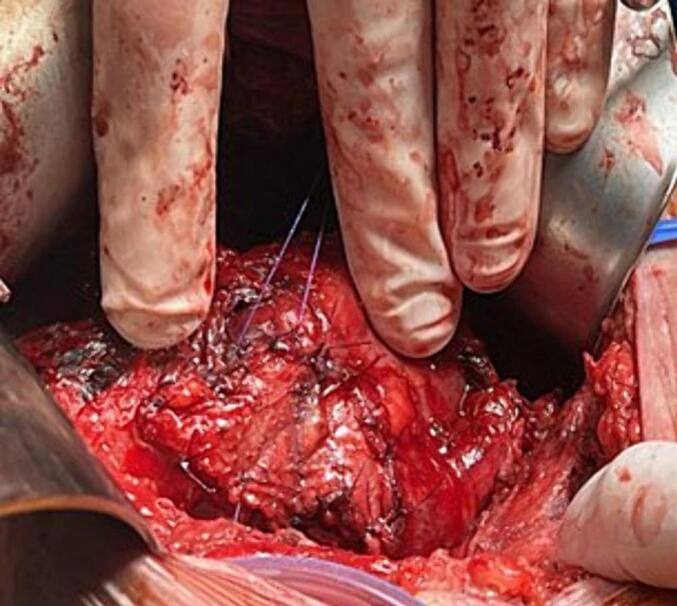
Intraoperative photo showing rectus sheath fascia patched over the pelvic defect, secured with 4.0 PDS interrupted sutures.

During reoperation, a focal 1 cm × 1.5 cm area of superior renal pelvic necrosis was observed (Fig. 1). The surrounding tissue appeared well vascularised. The proximal and distal ureter was healthy and intact. Via the indwelling urinary catheter, methylene blue stained 0.9 % sodium chloride solution was infused into the bladder. Extravasation was noted at the renal pelvis, but no leak was identified at either the ureteric anastomosis or bladder. A decision was made to reconstruct the focal area of necrosis. The necrotic tissue was debrided back to healthy bleeding edges. A 3cm^2^ area of rectus sheath fascia was excised and patched onto the open pelvic defect (Fig. 2). The patch was secured with interrupted 4.0 polydioxanone sutures (PDS). Given acute cellular or antibody mediated rejection can increase the risk of a urine leak, a concurrent open biopsy was performed however it did not show any evidence of rejection.

**Fig. 3:**
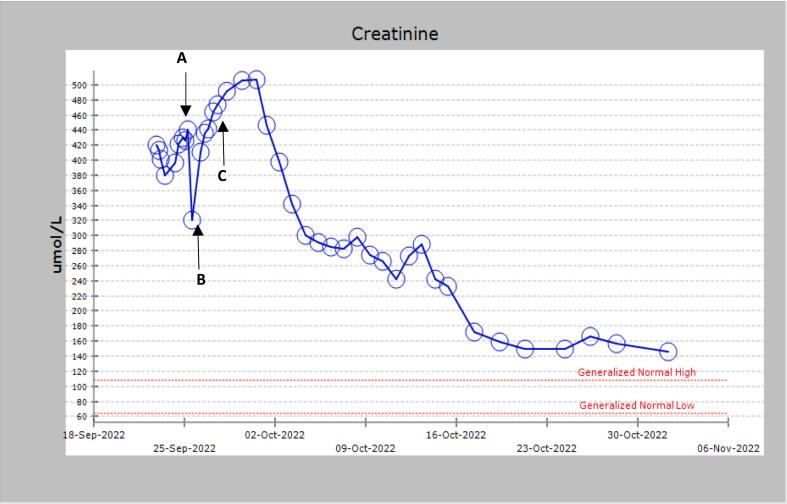
Graph demonstrating creatinine trend over three months. **(A)** Preoperative creatinine, **(B)** Day 2 post renal transplant, **(C)** day 3 post renal transplant following discovery of urine leak with associated return to theatre.

Following operative intervention, serum creatinine decreased from 464 μmol/L to 131 μmol/L over a two-week period (Fig. 3). Drain output reduced to less than 50 mL/day with subsequent removal 18 days post-operatively**.** An indwelling catheter remained in-situ for 6 weeks. A 6-week post-operative retrograde pyelogram confirmed no urine leak. The patient was discharged from the acute transplant unit with regular nephrology follow-up. A 12-month post-operative ultrasound demonstrated a well perfused renal allograft without evidence of persisting urine leak or fluid collection. The patient's creatinine remained stable 18 months post transplant.

## 3 Discussion

The incidence of urine leakage following kidney transplantation range between 1.5 and 8.9 % [2]. Devascularisation and subsequent necrosis of the urinary system including ureter are the most common causes. The transplanted renal pelvis receives its blood supply exclusively from the renal artery via smaller branches directed longitudinally, transversely and obliquely within the peripelvic fat. Extensive dissection, accidental ligation, or denudation of these structures during organ recovery or back table preparation increase the risk for isolated necrosis. Additional risk factors include increased cold and warm ischaemic time, rejection episodes, and infection [4,5]. However, the cause of renal pelvic necrosis in the aforementioned case remains unknown.

Average waiting times in Australia for renal transplantation are between three and five years [9]. Given the shortage of donor organs, every implanted graft must, if possible be preserved. Graft nephrectomy carries not only surgical risk but increased immunological risk due to pre-sensitisation of donor antigens making a second renal transplant more complex. Given the isolated focal renal pelvis necrosis in an otherwise well perfused functioning graft surgical reconstruction was justifiable. In an attempt to restore anatomy, the pelvic defect was patched with avascular rectus sheath fascia. Fascia was used as a template bridge allowing scar tissue formation to reinforce the repair. Advantages of this reconstructive method were technique simplicity and low donor site morbidity. Potential complications included patch failure with ongoing urine leak, ventral wall hernia through the fascial donor site and stenosis of the ureteropelvic junction. A 6-week post-operative retrograde pyelogram confirmed no ongoing urine leak, a baseline serum creatinine of 131 μmol/L was achieved, and the patient reported a good quality of life following removal of the indwelling urinary catheter.

Given the isolated necrosis localised to the renal pelvis, standard open surgical approaches such as ureteric reimplantation or reconstructive pyeloureterostomy with native ureter were deemed inappropriate. While surgery for distal and long segment ureteric reconstruction is well reported, this is, to our knowledge, the second described case of attempted surgical reconstruction of the transplanted renal pelvis. Schlitt and Hoffman previously described reconstruction of the transplanted renal pelvis using a vascularised small bowel patch. However, this technique was complex, suffered from recurrent urinary leaks requiring reoperation and nephrostomy tube, and there were multiple urinary tract infections [10]. In contrast, the avascular rectus sheath patch was chosen as it was technically simple, had low potential donor site morbidity and allowed the peritoneal cavity to remain intact.

## 4 Conclusion

This case highlights the successful surgical management of a renal pelvis urine leak patched with rectus sheath fascia. This novel technique could be considered as a graft saving procedure in similar cases where the alternative is transplant nephrectomy.

## Consent

Written informed consent was obtained from the patient for publication of this case report and accompanying images. A copy of the written consent is available for review by the Editor-in-Chief of this journal on request.

## Ethical approval

Ethics was obtained from the Metro South Health Service. Project ID 79773. Project Title: Rectus sheath patch. A novel surgical technique in the repair of isolated renal pelvis necrosis in a transplanted kidney. Review Reference: HREC/2023/QMS/79773.

## Funding

This research did not receive aby specific grant from funding agencies in the public, commercial, or not-for-profit sectors.

## Author contribution

Dr. Sebastian Primrose: Written the paper.

Dr. Ai Lin Tan: Manuscript Editor.

Dr. Malcolm Lawson: Manuscript Editor.

Dr. Handoo Rhee: Manuscript Editor.

Dr. Anthony Griffin: Manuscript Editor.

## Guarantor

Dr. Sebastian Primrose.

## Research registration number

Not applicable. Not a first in man clinical trial.

## Conflict of interest statement

The authors of this manuscript have no conflicts of interest to disclose.
